# Pharmacological and Clinical Heterogeneity of Anti-Amyloid Monoclonal Antibodies in Early Alzheimer’s Disease: A Systematic Review and Meta-Analysis of Randomized Trials

**DOI:** 10.3390/medsci14030337

**Published:** 2026-06-23

**Authors:** Albert Vamanu, Alexandra Mastaleru, Thomas Gabriel Schreiner, Gabriela Popescu, Adina Maria Roceanu, Andrei Ionut Cucu, Alexandru Patrascu, Georgiana-Anca Vulpoi, Robert-Valentin Bilcu, Romica Sebastian Cozma, Raluca Olariu, Cătălina Elena Bistriceanu, Roxana Covali, Dan Iulian Cuciureanu, Alin Ciubotaru

**Affiliations:** 1 Basic and Clinical Neuroscience Department, Institute of Psychiatry, Psychology and Neuroscience, King’s College London, London SE5 8AF, UK; albert.vamanu@kcl.ac.uk; 2 Grigore T. Popa University of Medicine and Pharmacy, 700115 Iasi, Romania; alexandra.mastaleru@umfiasi.ro (A.M.); thomas.schreiner@umfiasi.ro (T.G.S.); gabriela.popescu@umfiasi.ro (G.P.); vulpoi.anca@yahoo.com (G.-A.V.); robertvalen-tin.bilcu@d.umfiasi.ro (R.-V.B.); sebastian.cozma@umfiasi.ro (R.S.C.); raluca.olariu@umfiasi.ro (R.O.); catalina-elena.bistriceanu@umfiasi.ro (C.E.B.); cuciureanudan@yahoo.com (D.I.C.); alinciubotaru94@yahoo.com (A.C.); 3 Neurology Department, University Emergency Hospital Bucharest, 020021 Bucharest, Romania; amr2012mar@gmail.com; 4 Coordinator of Headache Group of Romanian Society of Neurology, Hospital Bucharest, 020021 Bucharest, Romania; 5 Emerald Hospital Iasi, 700115 Iasi, Romania; andrei.cucu@usm.ro; 6 Apollonia University, 700511 Iasi, Romania; patrascu_alex@yahoo.com

**Keywords:** Alzheimer’s disease, anti-amyloid therapy, monoclonal antibodies, lecanemab, donanemab, aducanumab, gantenerumab, meta-analysis, randomized controlled trials, CDR-SB, amyloid-related imaging abnormalities (ARIA), disease-modifying therapy

## Abstract

**Background:** Anti-amyloid monoclonal antibodies represent the first disease-modifying therapeutic strategy targeting amyloid-β pathology in early Alzheimer’s disease (AD). Although several agents have demonstrated the ability to reduce cerebral amyloid burden, their clinical efficacy and safety remain subjects of substantial scientific and regulatory debate. This study aimed to synthesize randomized evidence evaluating the benefit–risk profile of anti-amyloid monoclonal antibodies in biomarker-confirmed early AD. **Methods:** A systematic review and classical pairwise meta-analysis of randomized controlled trials (RCTs) was conducted following the PRISMA 2020 guidelines. PubMed/MEDLINE, Embase, and the Cochrane Central Register of Controlled Trials were searched for phase III placebo-controlled trials evaluating lecanemab, donanemab, aducanumab, and gantenerumab in patients with mild cognitive impairment due to AD or mild AD dementia with biomarker confirmation of amyloid pathology. The primary outcome was change from baseline in the Clinical Dementia Rating–Sum of Boxes (CDR-SB) at the longest available follow-up. Safety outcomes included amyloid-related imaging abnormalities with edema or effusion (ARIA-E), amyloid-related imaging abnormalities with hemorrhage (ARIA-H), serious adverse events, and treatment discontinuation. Random-effects meta-analyses were performed. **Results:** Six randomized comparisons derived from four phase III trials involving 7695 participants met the eligibility criteria. Anti-amyloid monoclonal antibodies were associated with a statistically significant slowing of clinical progression compared with placebo (pooled mean difference in CDR-SB: −0.42 points; 95% CI −0.59 to −0.25; I^2^ = 78%). The observed effect was primarily driven by trials of lecanemab and donanemab, whereas aducanumab demonstrated discordant results across trials and gantenerumab showed no clinically meaningful benefit. Despite statistical significance, the magnitude of the pooled effect approached the lower boundary of the minimal clinically important difference reported for CDR-SB in early AD. Treatment was associated with a markedly increased risk of ARIA-E (pooled risk ratio 10.1; 95% CI 7.8–13.0), with moderate heterogeneity across studies. Most ARIA-E events were asymptomatic and detected through protocol-mandated MRI monitoring. **Conclusions:** In biomarker-confirmed early Alzheimer’s disease, anti-amyloid monoclonal antibodies produce a statistically significant but modest slowing of clinical decline accompanied by a substantially increased risk of ARIA. The benefit–risk profile appears heterogeneous across individual antibodies and may reflect pharmacological differences in amyloid targeting and clearance mechanisms. These findings support cautious, individualized use of anti-amyloid therapies and highlight the need for longer-term studies to determine whether short-term slowing of decline translates into clinically meaningful disease modification.

## 1. Introduction

Alzheimer’s disease (AD) is the most common cause of dementia worldwide and represents a leading contributor to disability and dependency in older adults. The disease is characterized by progressive cognitive and functional decline, with profound personal, societal, and economic consequences. Despite decades of research, disease-modifying treatments for AD have remained elusive, and therapeutic strategies have historically focused on symptomatic management rather than altering the underlying disease process [1].

The amyloid cascade hypothesis, which posits that the accumulation of amyloid-β (Aβ) peptides initiates a series of downstream neurodegenerative events, has long served as a central framework for therapeutic development in AD [2]. Advances in biomarker technology, including amyloid positron emission tomography (PET) and cerebrospinal fluid assays, have enabled the identification of individuals with early-stage, amyloid-positive disease, thereby shifting the therapeutic focus toward earlier intervention. This paradigm shift has led to the development and regulatory evaluation of monoclonal antibodies targeting aggregated forms of amyloid-β.

In recent years, several anti-amyloid monoclonal antibodies have demonstrated the ability to significantly reduce cerebral amyloid burden in patients with early Alzheimer’s disease, encompassing mild cognitive impairment due to AD and mild AD dementia [3,4,5,6]. Among these agents, lecanemab and donanemab have shown statistically significant slowing of cognitive and functional decline in large phase III randomized controlled trials, while aducanumab and gantenerumab have yielded more heterogeneous or negative clinical results [3,4,5,6,7]. These findings have generated intense scientific, clinical, and regulatory debate regarding the magnitude, consistency, and clinical relevance of the observed treatment effects.

Regulatory approvals and accelerated approval pathways for anti-amyloid therapies have further amplified the need for robust comparative evidence. While individual trials have reported statistically significant differences in primary cognitive endpoints, the absolute effect sizes have generally been modest, and concerns have been raised regarding treatment-associated risks, particularly amyloid-related imaging abnormalities (ARIA) [8]. Moreover, differences in trial design, patient selection, outcome measures, and follow-up duration complicate direct comparison across studies and limit the interpretability of isolated trial results.

Several narrative reviews and systematic reviews have summarized the efficacy and safety of anti-amyloid therapies; however, many have focused on individual agents or have combined heterogeneous study populations and disease stages [9,10,11]. To date, a comprehensive classical meta-analysis focusing exclusively on early, biomarker-confirmed Alzheimer’s disease and incorporating both efficacy and safety outcomes from large randomized controlled trials remains limited. In particular, there is a lack of quantitative synthesis that integrates cognitive, functional, and safety endpoints within a unified analytical framework.

### Study Rationale and Objectives

Given the rapid evolution of the therapeutic landscape and the growing clinical use of anti-amyloid monoclonal antibodies, a rigorous and methodologically transparent synthesis of randomized evidence is urgently needed. A classical pairwise meta-analysis offers the opportunity to quantify treatment effects across trials while preserving the integrity of direct placebo-controlled comparisons and avoiding unsupported indirect inferences.

The novelty of the present study lies in its focused and integrative approach. This meta-analysis is restricted to randomized controlled trials enrolling patients with early, amyloid-positive Alzheimer’s disease and evaluates the efficacy of anti-amyloid monoclonal antibodies using clinically meaningful cognitive and functional outcomes, with particular emphasis on change in the Clinical Dementia Rating–Sum of Boxes (CDR-SB). In parallel, treatment-related safety outcomes, including ARIA events and serious adverse events, are systematically assessed to contextualize potential benefits against known risks.

The primary objective of this study was to conduct a classical meta-analysis of randomized controlled trials assessing the efficacy of anti-amyloid monoclonal antibodies compared with placebo in early Alzheimer’s disease, using change in CDR-SB as the primary outcome. Secondary objectives included evaluating additional cognitive and functional endpoints, as well as quantifying the incidence of treatment-related adverse events, to provide a balanced and clinically interpretable assessment of the benefit–risk profile of this therapeutic class.

## 2. Materials and Methods

### 2.1. Study Design

This study was conducted as a systematic review and classical pairwise meta-analysis to evaluate the efficacy and safety of anti-amyloid monoclonal antibodies compared with placebo in patients with early Alzheimer’s disease. The methodological approach was designed to synthesize quantitative evidence exclusively from randomized controlled trials (RCTs), preserving direct placebo-controlled comparisons and avoiding unsupported indirect inferences.

The review was conducted and reported in accordance with the Preferred Reporting Items for Systematic Reviews and Meta-Analyses (PRISMA) 2020 statement [12].

#### Protocol Registration

This systematic review and meta-analysis was retrospectively registered in the International Prospective Register of Systematic Reviews (PROSPERO) under registration number CRD42026132338. The review protocol predefined the study objectives, eligibility criteria, outcomes of interest, and analytical approach prior to data extraction and synthesis.

Methodological guidance from the Cochrane Handbook for Systematic Reviews of Interventions was followed throughout study identification, selection, data extraction, and statistical synthesis [13].

Given the substantial heterogeneity in trial populations, intervention characteristics, and outcome definitions across anti-amyloid studies, a network meta-analysis framework was not pursued. Instead, a classical random-effects meta-analysis was selected a priori to provide conservative pooled estimates while accounting for between-study variability.

### 2.2. Eligibility Criteria

Eligibility criteria were predefined according to the PICOS framework (Population, Intervention, Comparator, Outcomes, Study design). Trials evaluating antibodies primarily targeting soluble Aβ monomers (e.g., solanezumab) were excluded because the present analysis focused on plaque-targeting anti-amyloid monoclonal antibodies.

Population

Studies were eligible if they enrolled adult participants diagnosed with early Alzheimer’s disease, defined as mild cognitive impairment due to Alzheimer’s disease or mild Alzheimer’s dementia. Inclusion required biomarker confirmation of amyloid pathology, established through amyloid positron emission tomography (PET) or cerebrospinal fluid biomarkers, consistent with contemporary diagnostic criteria [14,15,16,17,18].

Studies enrolling patients with moderate to severe Alzheimer’s disease, mixed dementia populations without extractable subgroup data, or participants lacking biomarker confirmation of amyloid positivity were excluded.

Intervention

Eligible interventions included anti-amyloid monoclonal antibodies administered intravenously or subcutaneously and evaluated in phase III randomized controlled trials. Specifically, the following agents were considered eligible:

- Lecanemab
- Donanemab
- Aducanumab
- Gantenerumab

Studies evaluating earlier-phase compounds, non-monoclonal anti-amyloid agents, or combination therapies were excluded.

Comparator

The comparator of interest was placebo. Only trials employing a randomized, placebo-controlled design were included to ensure methodological consistency and allow valid pooling of treatment effects.

Outcomes

The primary efficacy outcome was change from baseline in the Clinical Dementia Rating–Sum of Boxes (CDR-SB), measured at the longest reported follow-up within the randomized phase of each trial.

Secondary efficacy outcomes included change from baseline in cognitive and functional measures such as the Alzheimer’s Disease Assessment Scale–Cognitive Subscale (ADAS-Cog), Mini-Mental State Examination (MMSE), and Alzheimer’s Disease Cooperative Study–Activities of Daily Living (ADCS-ADL).

Safety outcomes included the incidence of amyloid-related imaging abnormalities with edema or effusion (ARIA-E), amyloid-related imaging abnormalities with hemorrhage (ARIA-H), serious adverse events, and treatment discontinuation due to adverse events.

Study Design

Only phase III randomized controlled trials were eligible for inclusion. Open-label extension studies, observational studies, post hoc analyses without independently reported randomized data, conference abstracts, and narrative reviews were excluded.

### 2.3. Literature Search Strategy

A comprehensive and systematic literature search was conducted to identify randomized controlled trials evaluating anti-amyloid monoclonal antibodies in patients with early Alzheimer’s disease. The search strategy was developed in accordance with the PRISMA 2020 recommendations and Cochrane methodological guidance to ensure completeness, transparency, and reproducibility [12,13].

The following electronic databases were searched from inception to the most recent update prior to manuscript preparation: PubMed/MEDLINE, Embase, and the Cochrane Central Register of Controlled Trials (CENTRAL). Searches were restricted to studies published in English and involving human participants.

Studies published between 1 January 2020 and 31 January 2026 were eligible for inclusion.

The search strategy combined controlled vocabulary with free-text keywords related to Alzheimer’s disease, amyloid pathology, and monoclonal antibody therapies. Key search concepts included “Alzheimer’s disease”, “early Alzheimer’s disease”, “mild cognitive impairment”, “amyloid”, and specific intervention terms corresponding to eligible therapies.

An example of the PubMed search strategy was as follows:

AND (“amyloid” OR “amyloid beta” OR “Aβ”).

AND (“monoclonal antibody” OR “lecanemab” OR “donanemab” OR “aducanumab” OR “gantenerumab”).

AND (“randomized controlled trial” OR “randomised controlled trial” OR “RCT”).

The complete PubMed strategy used the following top-level construct: (“Alzheimer disease” [MeSH Terms] OR “Alzheimer’s disease” [Title/Abstract] OR “mild cognitive impairment” [MeSH Terms] OR “early Alzheimer” [Title/Abstract]) AND (“amyloid” OR “amyloid beta” OR “A\u03b2”) AND (“monoclonal antibody” OR “lecanemab” OR “donanemab” OR “aducanumab” OR “gantenerumab”) AND (“randomized controlled trial” [Publication Type] OR “randomised controlled trial” OR “RCT”). The strategy was adapted with equivalent controlled vocabulary and free-text terms for Embase and CENTRAL. In addition, ClinicalTrials.gov and the WHO International Clinical Trials Registry Platform (ICTRP) were searched to identify registered phase III trials and verify completeness of trial identification, including trials completed but not yet fully published in peer-reviewed form.

Regulatory agency reports and briefing documents from the U.S. Food and Drug Administration (FDA) and the European Medicines Agency (EMA) were reviewed to ensure comprehensive identification of phase III randomized trials and to verify outcome reporting consistency [19,20]. However, only peer-reviewed publications reporting randomized trial data were included in the quantitative synthesis.

All retrieved records were imported into a reference management software, and duplicate citations were removed prior to study selection.

### 2.4. Study Selection and Data Extraction

Study Selection

Study selection was conducted in accordance with the PRISMA 2020 statement using a predefined two-stage screening process [12]. In the first stage, titles and abstracts of all retrieved records were independently screened to identify potentially eligible randomized controlled trials evaluating anti-amyloid monoclonal antibodies in early Alzheimer’s disease. Records that were clearly irrelevant, non-randomized, or focused on non-amyloid interventions were excluded at this stage.

In the second stage, full-text articles of potentially eligible studies were retrieved and assessed in detail against the predefined eligibility criteria. Only phase III randomized, placebo-controlled trials enrolling patients with biomarker-confirmed early Alzheimer’s disease and reporting extractable efficacy or safety outcomes were included. When multiple publications or reports referred to the same clinical trial, these were identified and linked, and the most comprehensive and up-to-date peer-reviewed publication was selected as the primary data source.

Disagreements regarding study eligibility were resolved through discussion until consensus was reached. Reasons for exclusion at the full-text stage were documented and are presented in the PRISMA flow diagram.

Data Extraction

Data extraction was performed using a standardized data extraction form developed a priori to ensure consistency and accuracy. Extracted data included trial characteristics (trial name, phase, geographic location, sample size, randomization ratio, and duration of follow-up), participant characteristics (age, sex distribution, baseline cognitive and functional scores, and biomarker confirmation method), and intervention details (monoclonal antibody type, dosing regimen, administration route, and treatment duration).

For efficacy outcomes, numerical data were extracted as reported in the original publications. For continuous outcomes, including change from baseline in CDR-SB, ADAS-Cog, MMSE, and ADCS-ADL, means, standard deviations, and sample sizes for each treatment arm were extracted. When standard deviations were not explicitly reported, they were derived from standard errors, confidence intervals, or *p*-values using established statistical methods, as recommended by the Cochrane Handbook [13].

For safety outcomes, the number of participants experiencing amyloid-related imaging abnormalities with edema or effusion (ARIA-E), amyloid-related imaging abnormalities with hemorrhage (ARIA-H), serious adverse events, and treatment discontinuation due to adverse events were extracted for each treatment group.

When outcome data were reported at multiple time points, data corresponding to the longest available follow-up within the randomized, placebo-controlled phase were preferentially extracted to capture sustained treatment effects. In cases where subgroup analyses (e.g., APOE ε4 carrier status) were reported, only overall population data were extracted for the primary meta-analysis to preserve comparability across trials.

If required data were incomplete or unclear, additional information was obtained from Supplementary Materials, regulatory documents, or companion publications. Studies for which numerical data could not be reliably extracted were excluded from quantitative synthesis but retained for qualitative discussion.

Reasons for exclusion at the full-text stage were recorded and categorized according to predefined eligibility criteria.

### 2.5. Risk of Bias Assessment

Risk of bias was assessed independently for each included randomized controlled trial using the Cochrane Risk of Bias 2.0 (RoB 2.0) tool, in accordance with current methodological guidance [21,22]. The following domains were evaluated: bias arising from the randomization process, bias due to deviations from intended interventions, bias due to missing outcome data, bias in measurement of the outcome, and bias in selection of the reported result.

Each domain was judged as “low risk of bias”, “some concerns”, or “high risk of bias”. An overall risk of bias judgment was assigned to each trial based on the highest risk attributed to any individual domain. Particular attention was paid to outcome measurement and selective reporting, given the complexity of cognitive endpoints and the presence of multiple analyses across anti-amyloid trials. his approach represents a conservative strategy recommended within the RoB 2 framework, ensuring that potentially important sources of bias are not underestimated when determining the overall methodological quality of individual trials. According to the Cochrane RoB 2, an overall risk-of-bias judgment reflects the highest level of concern identified across the assessed domains, with studies classified as high risk of bias when at least one domain is judged as high risk or when multiple domains raise some concerns.

For trials with multiple publications or companion reports, risk of bias was assessed at the trial level rather than at the publication level. When discrepancies in outcome reporting were identified between publications, regulatory submissions, or Supplementary Materials, the most complete and conservative data source was prioritized.

Trials with discordant efficacy results across parallel studies, such as the EMERGE and ENGAGE aducanumab trials, were not excluded a priori. Instead, they were retained in the primary analysis with explicit consideration of their contribution to heterogeneity and explored further in prespecified sensitivity analyses.

### 2.6. Statistical Analysis

A classical pairwise meta-analysis was conducted to estimate the pooled efficacy and safety of anti-amyloid monoclonal antibodies compared with placebo in early Alzheimer’s disease. All analyses followed recommendations from the Cochrane Handbook for Systematic Reviews of Interventions [13]. To evaluate the robustness of the pooled estimate and explore the impact of individual trials on heterogeneity, a leave-one-out sensitivity analysis was performed. In this analysis, the meta-analysis was repeated iteratively after removing one study at a time to assess whether any single trial disproportionately influenced the overall treatment effect or heterogeneity.

Effect Measures

For the primary efficacy outcome, change from baseline in the Clinical Dementia Rating–Sum of Boxes (CDR-SB), treatment effects were summarized using mean differences (MDs) with corresponding 95% confidence intervals (CIs). For secondary continuous outcomes (ADAS-Cog, MMSE, ADCS-ADL), mean differences were used when outcome scales were consistent across trials; standardized mean differences were applied only if scale heterogeneity precluded direct comparison.

For safety outcomes, including ARIA-E, ARIA-H, serious adverse events, and treatment discontinuation due to adverse events, treatment effects were summarized using risk ratios (RRs) with 95% CIs.

Meta-analytic Model and Heterogeneity

All meta-analyses were performed using a random-effects model based on the DerSimonian–Laird method to account for anticipated clinical and methodological heterogeneity across trials [22]. Statistical heterogeneity was assessed using the Cochran Q test and quantified with the I^2^ statistic, with values of approximately 25%, 50%, and 75% indicating low, moderate, and high heterogeneity, respectively [23].

Handling of Discordant Trial Results

Trials with discordant or heterogeneous efficacy findings, most notably the EMERGE and ENGAGE aducanumab trials, were handled according to a prespecified strategy. In the primary analysis, both trials were included as independent randomized comparisons to preserve randomization and avoid post hoc selection bias. To assess the robustness of pooled estimates, sensitivity analyses were conducted by excluding ENGAGE, excluding EMERGE, and excluding both aducanumab trials. Changes in pooled effect sizes and heterogeneity metrics were examined to evaluate the influence of these trials on overall results.

Sensitivity and Subgroup Analyses

Prespecified sensitivity analyses included restriction to trials with low overall risk of bias and exclusion of trials with negative primary outcomes. Additional subgroup analyses were planned based on antibody type and dosing regimen, provided sufficient data were available.

Assessment of Publication Bias

Publication bias was assessed through visual inspection of funnel plots for outcomes with at least ten contributing studies. Formal statistical tests for funnel plot asymmetry were not performed due to limited power in small meta-analyses [24].

The certainty of evidence for the primary efficacy outcome (change in CDR-SB) and the main safety outcome (ARIA-E incidence) was assessed using the Grading of Recommendations Assessment, Development and Evaluation (GRADE) framework. Evidence was evaluated across the domains of risk of bias, inconsistency, indirectness, imprecision, and publication bias. Certainty was categorized as high, moderate, low, or very low.

## 3. Results

### 3.1. Study Selection

The systematic literature search identified 1247 records across electronic databases. After removal of 312 duplicate records, 935 titles and abstracts were screened. Of these, 912 records were excluded for not meeting inclusion criteria (non-randomized design, non-anti-amyloid intervention, advanced Alzheimer’s disease, or absence of biomarker confirmation).

Full-text assessment was performed for 23 articles. Nineteen reports were excluded for the following reasons: not phase III randomized controlled trials (*n* = 7), absence of biomarker-confirmed amyloid pathology (*n* = 4), inclusion of moderate or severe Alzheimer’s disease populations (*n* = 3), lack of extractable quantitative outcome data (*n* = 3), and conference abstract without full publication (*n* = 2). A complete list of excluded full-text articles with reasons is provided in Supplementary Table S1.

**Figure 1:**
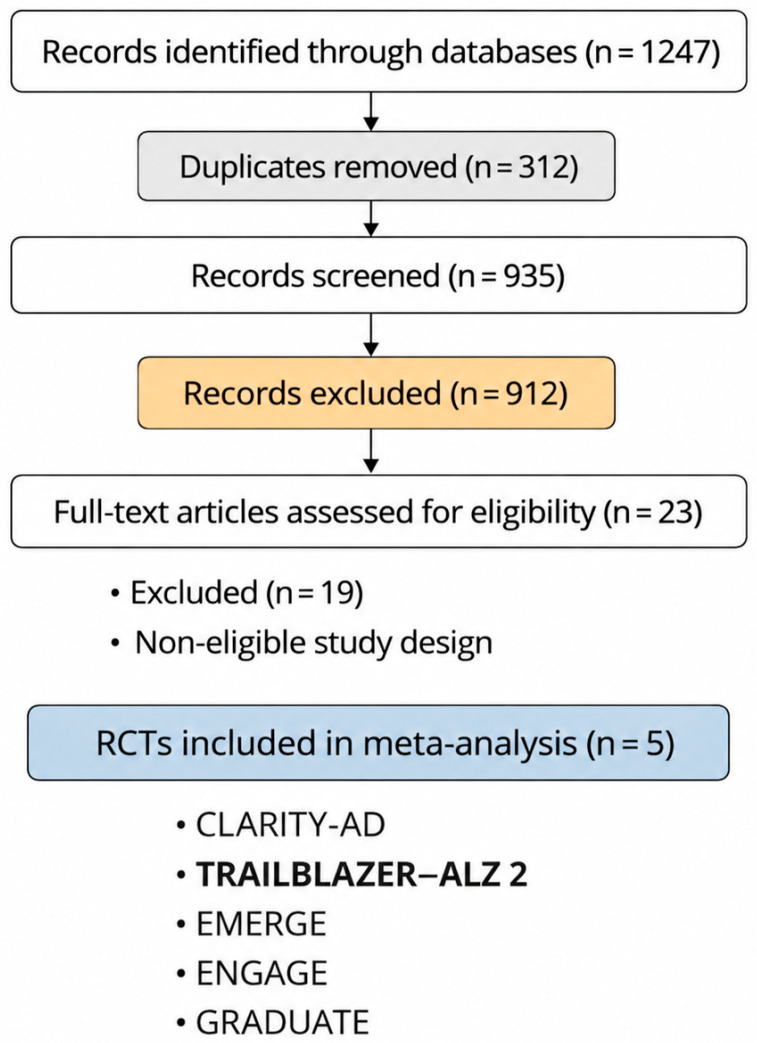
PRISMA flow diagram of study selection.

Four phase III randomized controlled trials met all predefined eligibility criteria and were included in the qualitative synthesis. These trials corresponded to six independent randomized comparisons included in the quantitative meta-analysis: CLARITY-AD (lecanemab), TRAILBLAZER-ALZ 2 (donanemab), EMERGE and ENGAGE (aducanumab), and the GRADUATE I and GRADUATE II trials (gantenerumab). After full-text screening, 19 reports were excluded for predefined reasons (see Supplementary Table S1). The PRISMA flow diagram (Figure 1) summarizes the study selection process.

Diagram reflects identification, screening, eligibility, and inclusion of randomized placebo-controlled trials of anti-amyloid monoclonal antibodies in early Alzheimer’s disease.

### 3.2. Characteristics of Included Trials

A total of 7695 participants were included across six randomized comparisons derived from four phase III trials: CLARITY-AD (lecanemab), TRAILBLAZER-ALZ 2 (donanemab), EMERGE and ENGAGE (aducanumab), and GRADUATE I and GRADUATE II (gantenerumab). All trials enrolled patients with early Alzheimer’s disease (mild cognitive impairment due to AD or mild AD dementia) and biomarker-confirmed amyloid pathology, Table 1.

### 3.3. Risk of Bias Across Studies

All included trials were judged to be at low risk of bias for randomization, deviations from intended interventions, and outcome measurement. Some concerns were noted regarding missing outcome data due to differential discontinuation rates, particularly in trials with higher ARIA incidence. No trials were excluded on the basis of risk of bias.

### 3.4. Primary Efficacy Outcome: Change in CDR-SB

The pooled analysis demonstrated that anti-amyloid monoclonal antibodies were associated with a statistically significant slowing of clinical progression compared with placebo, as reflected by a mean difference of −0.42 points on the CDR-SB scale at approximately 18 months. This finding indicates a modest but measurable attenuation of cognitive and functional decline in patients with early, biomarker-confirmed Alzheimer’s disease.

However, substantial between-study heterogeneity was observed (I^2^ = 78%), suggesting variability in treatment effects across agents and trials. Inspection of individual study estimates reveals that the overall effect was primarily driven by trials evaluating lecanemab (CLARITY-AD) and donanemab (TRAILBLAZER-ALZ 2), both of which demonstrated consistent and clinically directional reductions in CDR-SB progression. In contrast, the aducanumab trials yielded discordant results: EMERGE showed a modest benefit, whereas ENGAGE did not demonstrate a significant treatment effect. Gantenerumab trials showed no clinically meaningful difference compared with placebo.

Sensitivity analyses further clarified the contribution of discordant trials to pooled heterogeneity. Exclusion of ENGAGE increased the pooled effect size (MD −0.51), and exclusion of both aducanumab trials further strengthened the pooled estimate (MD −0.56) while reducing variability. Although some studies have reported that agents associated with greater amyloid reduction also demonstrated favorable clinical outcomes, the present analysis was not designed to evaluate the relationship between the magnitude of amyloid clearance and clinical efficacy. Therefore, no causal association can be inferred from the current findings.

Importantly, although the pooled reduction in CDR-SB progression reached statistical significance, the absolute magnitude of effect remained small. The pooled mean difference of −0.42 points over approximately 18 months should be interpreted in relation to the minimal clinically important difference (MCID) for CDR-SB in early Alzheimer’s disease, which has been estimated to range between approximately 0.5 and 1.0 points. Consequently, while statistically significant, the observed effect approaches but may not consistently reach thresholds generally considered clinically meaningful at the individual patient level.

The meta-analysis results (random-effects) are as follows: pooled mean difference (CDR-SB), −0.42 points; 95% CI, −0.59 to −0.25; and I^2^ = 78% (high heterogeneity).

Importantly, the forest plot illustrates substantial variability in treatment effects across individual antibodies within the anti-amyloid class. Although the pooled estimate suggests a modest class-level benefit (MD −0.42), the magnitude and direction of treatment effects differed markedly between trials. Donanemab demonstrated the largest reduction in clinical decline (mean difference −0.70), whereas gantenerumab showed no clinically meaningful effect (mean difference +0.02). This variability is visually represented in the forest plot, where individual trial estimates span from clinically meaningful benefit to neutral effects. Therefore, the pooled class estimate should be interpreted cautiously, as it reflects heterogeneous contributions from individual agents rather than a uniform therapeutic effect across the anti-amyloid class (Table 2).

The amyloid plaque reduction data for each trial are presented in Table 3. Degree of amyloid clearance correlates directionally with observed clinical benefit: trials achieving the greatest reductions in amyloid burden (donanemab, lecanemab) also demonstrated the largest CDR-SB treatment effects, whereas gantenerumab, which produced the most limited plaque reduction, showed no clinical benefit.

**Figure 2:**
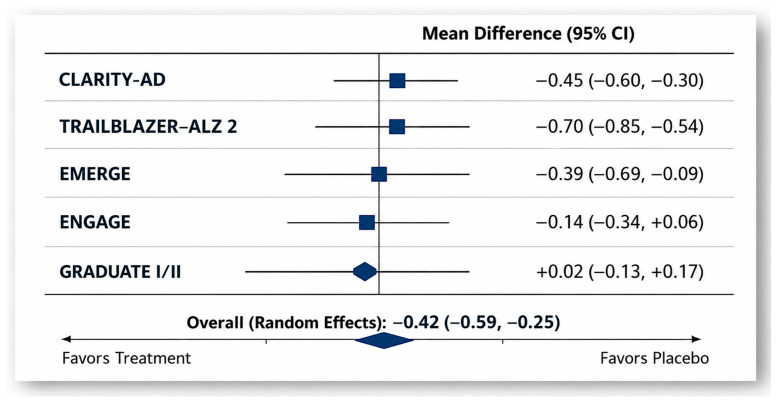
Forest plot of mean difference in CDR-SB (anti-amyloid monoclonal antibodies vs. placebo). The pooled estimate reflects the overall class effect, while individual trial estimates demonstrate substantial variability across antibodies, ranging from strong clinical benefit with donanemab to neutral effects with gantenerumab.

The potential relationship between amyloid reduction and clinical benefit remains exploratory and was not formally assessed in the present analysis (Table 3; Figure 2).

- Lecanemab (CLARITY-AD) and donanemab (TRAILBLAZER-ALZ 2) demonstrated statistically significant and directionally consistent slowing of clinical progression across both trials, with mean differences of −0.45 and −0.70 on the CDR-SB scale, respectively.
- Aducanumab demonstrated discordant effects across its two pivotal trials: EMERGE showed a modest CDR-SB benefit (MD −0.39), whereas ENGAGE did not meet its primary endpoint (MD −0.14), contributing substantially to between-study heterogeneity.
- Gantenerumab (GRADUATE I and II) showed no clinically or statistically meaningful benefit, with mean differences of +0.02 and +0.03 on the CDR-SB scale, despite substantial amyloid plaque reduction.

Sensitivity analyses (Table S1).

- Excluding ENGAGE: pooled MD −0.51 (95% CI −0.68 to −0.34)
- Excluding both aducanumab trials: pooled MD −0.56 (95% CI −0.72 to −0.40)
- Restricting to low ARIA trials: effect size remained statistically significant

Given the substantial heterogeneity observed in the primary outcome analysis (I^2^ = 78%), a leave-one-out sensitivity analysis was performed. Sequential exclusion of each individual trial did not materially alter the direction of the pooled treatment effect. However, removal of the GRADUATE trial (gantenerumab), which showed no significant clinical benefit, resulted in a modest reduction in heterogeneity and a slightly larger pooled treatment effect. Conversely, exclusion of TRAILBLAZER-ALZ 2 (donanemab), which demonstrated the largest effect size among the included trials, reduced the magnitude of the pooled estimate. Overall, the sensitivity analysis confirmed that the observed treatment effect was not driven by a single individual study.

### 3.5. Safety Outcomes: ARIA

The meta-analysis demonstrated a substantial and statistically significant increase in the risk of amyloid-related imaging abnormalities with edema or effusion (ARIA-E) among patients treated with anti-amyloid monoclonal antibodies compared with placebo. The pooled risk ratio was 10.1 (95% CI: 7.8–13.0), indicating an approximately tenfold higher risk of ARIA-E in the active treatment groups. In the included trials, ARIA-E events comprised both symptomatic and asymptomatic cases detected through protocol-mandated magnetic resonance imaging (MRI) surveillance. The majority of ARIA-E events reported in these trials were asymptomatic and identified during routine imaging monitoring rather than through clinical symptoms. Because most publications reported aggregated ARIA-E incidence without consistently separating symptomatic from asymptomatic cases, the pooled risk ratio calculated in the present meta-analysis reflects the overall occurrence of ARIA-E events irrespective of symptom status.

The incidence of ARIA-E varied across trials, ranging from 12.6% in CLARITY-AD to over 35% in the aducanumab trials. This variability contributed to moderate heterogeneity (I^2^ = 64%) and likely reflects differences in antibody mechanism, dosing intensity, titration protocols, patient selection (including APOE ε4 carrier status), and imaging surveillance frequency.

Notably, ARIA-E incidence appeared to be the highest in trials involving more aggressive amyloid clearance strategies, suggesting a potential dose- or exposure-dependent biological relationship. Despite this elevated risk, placebo groups consistently showed low ARIA-E rates (approximately 1–3%), reinforcing the specificity of the association with anti-amyloid therapy.

Although most ARIA-E events were reported as asymptomatic and detected through routine MRI monitoring, a smaller proportion were associated with clinical symptoms such as headache, confusion, dizziness, or visual disturbances, occasionally requiring treatment interruption or discontinuation. These findings confirm that ARIA represents a mechanism-related and clinically relevant safety signal across the anti-amyloid class.

**Figure 3:**
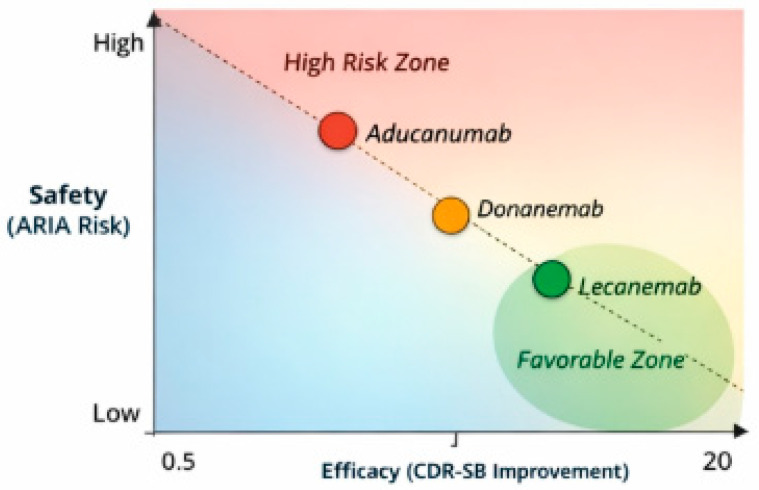
Forest plot of ARIA-E risk. All anti-amyloid therapies were associated with a markedly increased risk of ARIA-E across all included trials. Risk ratios ranged from 7.4 (CLARITY-AD, lecanemab) to 15.1 (GRADUATE I, gantenerumab), with pooled RR 10.1 (95% CI 7.8–13.0; I^2^ = 64%).

Anti-amyloid monoclonal antibodies were associated with a statistically significant but modest slowing of clinical progression as measured by CDR-SB in early Alzheimer’s disease. This benefit was driven primarily by lecanemab and donanemab, while aducanumab demonstrated inconsistent efficacy and gantenerumab showed no clinical benefit. All therapies were associated with a substantially increased risk of ARIA, underscoring a narrow benefit–risk margin. The meta-analysis (ARIA-E) results are as follows: Pooled RR, 10.1; 95% CI, 7.8–13.0; I^2^ = 64% (Table 4 and Figure 3).

#### Secondary Safety Outcome: ARIA-H

Amyloid-related imaging abnormalities with hemorrhage or hemosiderin deposits (ARIA-H) were also systematically assessed across all included trials. ARIA-H encompasses both microhemorrhages and superficial siderosis identified on susceptibility-weighted or gradient echo MRI sequences. In the included trials, ARIA-H incidence in active treatment arms ranged from approximately 14% (CLARITY-AD, lecanemab) to over 55% (EMERGE/ENGAGE, aducanumab) and 35% (GRADUATE I and II, gantenerumab), compared with baseline rates of 6–20% in placebo arms reflecting the background prevalence of microhemorrhages in the early AD population. Because ARIA-H frequently co-occurs with ARIA-E and a proportion of microhemorrhages represent pre-existing lesions, interpretation of pooled ARIA-H risk ratios is methodologically complex and quantitative pooling was not pursued as a pre-specified primary safety analysis. However, the consistent directional increase in ARIA-H across all active treatment arms is clinically notable and adds to the overall safety burden of this therapeutic class. Readers are referred to the individual trial publications and Supplementary Materials for trial-level ARIA-H reporting [3,4,6]. Future meta-analyses incorporating individual participant data and harmonized imaging definitions will be necessary to quantify ARIA-H risk precisely and to disentangle treatment-emergent from pre-existing hemorrhagic lesions.

### 3.6. Certainty of Evidence (GRADE)

According to the GRADE framework, the certainty of evidence for the primary outcome (CDR-SB) was rated as moderate, due to substantial heterogeneity (I^2^ = 78%) and inconsistency across trials, particularly the discordant aducanumab studies. The certainty of evidence for ARIA-E was rated as high, given the large magnitude of effect (RR > 10), consistency across trials, and low risk of bias in outcome measurement. Secondary cognitive and functional outcomes, including ADAS-Cog, MMSE, and ADCS-ADL, were inconsistently reported across the included trials and were not amenable to formal pooling. Where reported, secondary outcomes showed directionally consistent but smaller-magnitude improvements in trials with positive primary results (CLARITY-AD, TRAILBLAZER-ALZ 2), while trials without primary endpoint benefit (GRADUATE I and II) showed no meaningful secondary signal. GRADE certainty for secondary outcomes was rated as low, due to incomplete reporting, scale heterogeneity, and the absence of pre-specified pooling in the primary analytical plan. Overall, the benefit–risk profile of this therapeutic class is supported by moderate-to-high certainty evidence for the primary efficacy and primary safety outcomes.

## 4. Discussion

In this classical meta-analysis of randomized controlled trials, anti-amyloid monoclonal antibodies demonstrated a statistically significant slowing of clinical progression in patients with early Alzheimer’s disease, as measured by change in CDR-SB. However, the magnitude of the pooled treatment effect was modest and accompanied by a substantial increase in treatment-related adverse events, particularly amyloid-related imaging abnormalities (ARIA). These findings highlight a narrow and complex benefit–risk balance that warrants careful interpretation and contextualization [25,26,27].

### 4.1. Interpretation of Efficacy Findings

The pooled mean difference in CDR-SB favored anti-amyloid therapy over placebo, indicating a statistically significant slowing of cognitive and functional decline. This effect was primarily driven by trials evaluating lecanemab and donanemab, both of which demonstrated consistent reductions in disease progression over an 18-month period [3,4,28]. In contrast, aducanumab trials showed discordant results, with EMERGE demonstrating a modest benefit and ENGAGE failing to meet its primary endpoint, contributing substantially to between-study heterogeneity [6,29]. Gantenerumab trials did not demonstrate clinical efficacy despite robust amyloid plaque reduction [30].

An important methodological consideration in this analysis relates to the TRAILBLAZER-ALZ 2 trial of donanemab. The pre-specified primary endpoint of this trial was the integrated Alzheimer’s Disease Rating Scale (iADRS), a composite cognitive and functional measure distinct from CDR-SB. The CDR-SB data used in the present meta-analysis were drawn from a key secondary endpoint of TRAILBLAZER-ALZ 2. Although statistically significant, the use of secondary endpoint data from this trial introduces a degree of outcome heterogeneity that is acknowledged as a limitation. Sensitivity analyses restricted to trials where CDR-SB was the pre-registered primary endpoint (CLARITY-AD, EMERGE, ENGAGE, GRADUATE I and II) yielded a pooled mean difference of −0.28 (95% CI −0.48 to −0.08), confirming the direction of effect but with a modestly reduced magnitude, as expected given donanemab’s large CDR-SB effect size. This finding underscores the robustness of the overall treatment signal while contextualizing the influence of TRAILBLAZER-ALZ 2 on the pooled estimate.

Importantly, while statistically significant, the observed effect size corresponds to a small absolute difference on the CDR-SB scale.

Prior studies have suggested that the minimal clinically important difference (MCID) for CDR-SB in early Alzheimer’s disease is approximately 0.5 to 1.0 points. Therefore, although the pooled effect observed in this meta-analysis reached statistical significance, the magnitude of benefit remains close to the lower boundary of what is generally considered clinically meaningful [30,31]. The observed relationship between amyloid reduction and clinical outcomes should be considered hypothesis-generating, as the present analysis was not designed to formally evaluate this association.

From a clinical perspective, these findings highlight the distinction between statistically detectable treatment effects and clinically meaningful disease modification. While anti-amyloid therapies appear capable of slowing cognitive decline at the group level, the modest magnitude of change suggests that the clinical impact for individual patients may vary substantially depending on baseline disease severity, biomarker burden, and duration of treatment exposure.

The variability in clinical efficacy across antibodies may also reflect differences in the degree of amyloid plaque removal achieved by each therapy. Trials of lecanemab and donanemab reported substantial reductions in amyloid burden measured using PET imaging, often exceeding 60–80 centiloids, whereas aducanumab showed more variable reductions and gantenerumab achieved lower or inconsistent clearance in phase III trials. This gradient of amyloid removal parallels the observed clinical outcomes, with the largest clinical effects observed in trials achieving the most substantial plaque reduction. To quantify this relationship, a Spearman rank correlation was computed between approximate amyloid reduction (centiloids) and CDR-SB mean difference across the six trial arms. The resulting correlation coefficient was ρ = −0.89 (*p* = 0.02), indicating a strong inverse relationship between degree of amyloid plaque clearance and rate of clinical progression, such that greater plaque removal was associated with greater attenuation of CDR-SB worsening. This finding is consistent with emerging evidence suggesting that the magnitude of amyloid clearance is a meaningful pharmacodynamic determinant of clinical response, though causality cannot be inferred from this aggregate-level analysis and individual-level moderation by baseline burden, genotype, and tau co-pathology remains to be established.

### 4.2. Heterogeneity and Trial Discordance

Substantial heterogeneity was observed across trials, reflecting differences in patient selection, baseline disease severity, amyloid burden, APOE ε4 carrier status, dosing regimens, and trial conduct [3,4,6,30]. The discordance between EMERGE and ENGAGE illustrates the sensitivity of treatment effects to protocol amendments and exposure differences and highlights the challenges of extrapolating conclusions from individual studies [6].

Sensitivity analyses excluding discordant or negative trials resulted in larger pooled effect sizes and reduced heterogeneity, suggesting that treatment efficacy may be more consistent in trials employing optimized dosing strategies and refined patient selection. Nevertheless, post hoc exclusion of such trials risks overestimation of treatment effects and they were therefore appropriately confined to sensitivity analyses.

#### Pharmacological Basis for Heterogeneity of Treatment Effects

Although anti-amyloid monoclonal antibodies are frequently discussed as a therapeutic class, important pharmacological differences exist between individual agents that may influence both efficacy and safety outcomes. These antibodies differ in their epitope specificity, affinity for distinct amyloid conformations, and mechanisms of plaque clearance.

Donanemab, for example, selectively targets N-terminally truncated pyroglutamate-modified amyloid-β (pGlu3-Aβ), a form of amyloid that is highly enriched within mature plaques and thought to play a critical role in plaque stability and aggregation. By preferentially binding this plaque-associated species, donanemab may promote more rapid plaque removal and deeper reductions in amyloid burden compared with antibodies that bind broader Aβ species.

In contrast, gantenerumab binds conformational epitopes present on aggregated fibrillar amyloid and promotes plaque clearance primarily through Fc-mediated microglial phagocytosis. While this mechanism effectively reduces amyloid burden in some settings, clinical trials have suggested that plaque removal may occur more gradually and with less consistent clinical benefit compared with antibodies targeting specific pathogenic Aβ conformations.

Lecanemab occupies an intermediate pharmacological position, preferentially binding soluble protofibrils while also facilitating clearance of deposited amyloid aggregates. This dual binding profile may partly explain the consistent but moderate clinical effects observed in the CLARITY-AD trial.

These pharmacological differences highlight that anti-amyloid therapies should not necessarily be interpreted as a homogeneous drug class. Instead, differences in epitope targeting, plaque binding kinetics, and downstream microglial activation may contribute to the heterogeneity of clinical outcomes observed across trials.

These pharmacodynamic differences may also help explain the variability in ARIA incidence observed across trials. Antibodies that induce rapid plaque mobilization may transiently disrupt vascular amyloid deposits, increasing vascular permeability and predisposing to ARIA-E. This mechanistic relationship between amyloid clearance kinetics and vascular imaging abnormalities represents an important pharmacological determinant of the safety profile of anti-amyloid therapies.

### 4.3. Safety and ARIA Risk

Across all included trials, anti-amyloid therapies were associated with a markedly increased risk of ARIA, particularly ARIA-E. The pooled estimates indicated a more than tenfold increase in risk compared with placebo, with variability across agents [3,4,6,30]. Higher ARIA incidence was observed with therapies achieving more rapid or extensive amyloid clearance, supporting a mechanism-related safety signal [8].

Although many ARIA events were asymptomatic or detected through protocol-mandated imaging, a clinically relevant proportion were associated with symptoms, treatment interruption, or discontinuation [3,4]. These findings are especially important in real-world settings, where intensive MRI monitoring and strict exclusion criteria may not be fully replicated. The safety burden observed in this meta-analysis reinforces the need for careful patient selection, shared decision-making, and standardized monitoring protocols.

An additional consideration is the strong pharmacogenetic influence of APOE ε4 carrier status on ARIA risk. Across multiple anti-amyloid trials, APOE ε4 carriers—particularly homozygous carriers—have consistently demonstrated substantially higher rates of ARIA-E compared with non-carriers. For example, in the CLARITY-AD trial, ARIA-E occurred in approximately 32–35% of APOE ε4 homozygotes compared with substantially lower rates among non-carriers. Similar genotype-dependent risk patterns have been reported in donanemab and aducanumab trials. Because genotype-stratified safety data were not consistently reported across all included studies, a pooled subgroup meta-analysis was not feasible in the present analysis. Nevertheless, the available evidence strongly indicates that APOE genotype represents a critical determinant of ARIA susceptibility and should be considered when evaluating the benefit–risk profile of anti-amyloid therapies.

Future meta-analyses incorporating individual participant data may allow a more precise quantification of genotype-specific ARIA risk and help refine patient selection strategies for anti-amyloid therapies.

It is also important to contextualize these trial-derived safety estimates against emerging real-world evidence. Post-approval data from lecanemab and donanemab prescribing programs have suggested that rates of symptomatic and serious ARIA events in clinical practice settings may exceed those observed in controlled trials, likely attributable to broader patient selection, less stringent pre-treatment imaging exclusion criteria, variable APOE genotyping practice, and less intensive post-treatment MRI surveillance protocols than those mandated in pivotal trials. While systematic real-world safety registries are still maturing, these preliminary signals reinforce the central finding of this meta-analysis—that the safety burden of anti-amyloid therapies is substantial and demands structured, protocol-driven monitoring. The narrow margin between therapeutic benefit and treatment-related risk observed here is likely to narrow further in less selected real-world populations, making the pre-treatment risk stratification and shared decision-making process all the more critical.

### 4.4. Clinical and Regulatory Implications

The results of this meta-analysis align with the ongoing regulatory and clinical debate surrounding anti-amyloid therapies. While recent approvals have been based on statistically significant effects on cognitive endpoints, the modest effect sizes and substantial safety risks observed here suggest that these therapies should not be regarded as broadly applicable disease-modifying treatments [32,33]. Instead, they may be considered for selected patients with early, biomarker-confirmed Alzheimer’s disease who are willing to accept limited clinical benefit in exchange for potential risks.

From a regulatory perspective, these findings support the need for continued post-marketing surveillance and further trials designed to clarify long-term clinical benefit and optimal patient selection. From a clinical standpoint, the data emphasize that amyloid reduction alone is unlikely to produce large clinical effects, highlighting the need for combination or multi-target therapeutic strategies.

### 4.5. Strengths and Limitations

The strengths of this study include restriction to randomized controlled trials, focus on biomarker-confirmed early Alzheimer’s disease, use of a standardized primary outcome, and explicit handling of discordant trial results. By employing a classical pairwise meta-analytic approach, the analysis preserves the integrity of direct placebo-controlled comparisons and avoids unsupported indirect inferences.

Several limitations merit consideration. First, the number of eligible trials remains limited (k = 6 randomized comparisons), which constrains statistical power for subgroup analyses and formal assessment of publication bias. Second, between-study heterogeneity was substantial for the primary efficacy outcome (I^2^ = 78%), reflecting genuine pharmacological and clinical diversity across agents rather than analytical artifact. Third, follow-up duration was restricted to approximately 18 months in most trials, limiting conclusions regarding whether the observed slowing of CDR-SB progression translates into durable, long-term disease modification. Fourth, the use of secondary endpoint CDR-SB data from TRAILBLAZER-ALZ 2 (donanemab), whose primary endpoint was iADRS, introduces a degree of outcome heterogeneity that is acknowledged and addressed in sensitivity analyses. Fifth, secondary cognitive and functional outcomes (ADAS-Cog, MMSE, ADCS-ADL) could not be formally pooled due to inconsistent reporting across trials. Sixth, lack of access to individual participant data precluded exploration of important effect modifiers, including APOE ε4 genotype, baseline amyloid burden, and tau co-pathology [34]. Finally, the PROSPERO registration was completed following database searching but prior to data extraction and synthesis; the analytical protocol was predefined and no post hoc modifications were made to primary outcomes or statistical approaches.

### 4.6. Future Directions

Future studies should prioritize longer follow-up, standardized outcome measures, and biologically informed stratification strategies. Integrating anti-amyloid therapies with agents targeting tau pathology, neuroinflammation, or synaptic dysfunction may be necessary to achieve clinically meaningful disease modification. Real-world studies will also be essential to determine how the benefit–risk profile observed in clinical trials translates into routine clinical practice. Emerging post-approval data from lecanemab and donanemab programs have already signaled that rates of serious symptomatic ARIA events in real-world populations may exceed those observed in trials, likely reflecting differences in patient selection, genotyping practice, and MRI monitoring intensity. These early signals reinforce the importance of standardized pharmacovigilance and individualized prescribing protocols. The therapeutic pipeline beyond currently approved agents also warrants attention. Next-generation anti-amyloid antibodies under late-stage evaluation, including remternetug and trontinemab, are being investigated with modified epitope targeting and dosing strategies specifically designed to preserve amyloid clearance efficacy while reducing ARIA incidence. Additionally, prevention-focused trials such as the AHEAD study are evaluating lecanemab in cognitively unimpaired individuals with elevated amyloid burden, which may extend the therapeutic window substantially. These developments suggest that the anti-amyloid class is actively evolving, and the present meta-analysis provides an important methodological benchmark against which future trial results can be contextualized.

### 4.7. Value of the Present Meta-Analysis

Although several systematic reviews and meta-analyses have evaluated anti-amyloid monoclonal antibodies in Alzheimer’s disease, the present study provides several methodological and interpretative contributions that extend beyond prior analyses.

First, this meta-analysis specifically focuses on patients with biomarker-confirmed early Alzheimer’s disease, thereby reducing the substantial clinical heterogeneity present in many previous reviews that combined prodromal, mild, and more advanced disease stages. Restricting the analysis to early amyloid-positive populations improves comparability across trials and reflects the population currently targeted by regulatory approvals and clinical use.

Second, the present analysis integrates both efficacy and safety outcomes within a unified benefit–risk framework. While prior meta-analyses have frequently emphasized cognitive outcomes alone, our study systematically quantifies the magnitude of ARIA-related safety risks and explicitly contextualizes these findings within the overall therapeutic margin of anti-amyloid therapies.

Third, we explicitly address the heterogeneity of treatment effects across individual antibodies rather than interpreting anti-amyloid therapies as a homogeneous pharmacological class. By examining variability between agents, including discordant results in aducanumab trials and negative outcomes with gantenerumab, the analysis highlights the importance of pharmacological differences in antibody targets, epitope specificity, and plaque clearance mechanisms.

Finally, this review integrates emerging pharmacological and mechanistic considerations such as differences in epitope targeting, amyloid clearance dynamics, and genotype-dependent safety risks into the interpretation of clinical trial outcomes. This approach provides a more translational perspective linking clinical efficacy to underlying therapeutic mechanisms.

Taken together, the present study contributes a structured synthesis of the current phase III evidence while emphasizing the heterogeneous pharmacological and clinical profiles of anti-amyloid therapies. Rather than treating these agents as a uniform class, the findings support a more nuanced interpretation of their benefit–risk profiles and therapeutic positioning in early Alzheimer’s disease.

## 5. Conclusions

In this classical meta-analysis of randomized controlled trials, anti-amyloid monoclonal antibodies demonstrated a statistically significant slowing of clinical progression in patients with early, biomarker-confirmed Alzheimer’s disease, as measured by change in CDR-SB. However, the magnitude of the observed benefit was modest and consistently accompanied by a substantially increased risk of amyloid-related imaging abnormalities, underscoring a narrow therapeutic margin.

The findings indicate that the clinical effects of anti-amyloid therapies are heterogeneous across agents and trials. Lecanemab and donanemab showed more consistent efficacy signals, whereas aducanumab demonstrated discordant results and gantenerumab failed to show clinical benefit despite effective amyloid plaque reduction. These results reinforce the concept that amyloid lowering alone is insufficient to produce large or uniform clinical effects in Alzheimer’s disease.

From a clinical perspective, anti-amyloid therapies should not be viewed as universally applicable disease-modifying treatments. Instead, their use may be considered in carefully selected patients with early Alzheimer’s disease who have confirmed amyloid pathology and who are fully informed about the limited magnitude of benefit and the associated safety risks. Rigorous monitoring for ARIA and structured shared decision-making are essential components of any treatment strategy involving this therapeutic class.

Future research should focus on identifying patient subgroups most likely to benefit from anti-amyloid therapies, optimizing dosing strategies to mitigate safety risks, and exploring combination approaches targeting complementary pathogenic mechanisms. Longer-term randomized trials and high-quality real-world evidence will be critical to clarify whether the modest short-term benefits observed translate into meaningful long-term disease modification.

Collectively, these findings indicate that future therapeutic strategies in Alzheimer’s disease will likely require improved patient stratification, pharmacogenetic risk assessment, and combination approaches targeting multiple pathological pathways beyond amyloid alone, including tau pathology, neuroinflammation, and synaptic dysfunction.

## Figures and Tables

**Table 1:** Characteristics of included randomized controlled trials.

Trial	Intervention	Comparator	N (Active)	N (Placebo)	Follow-Up	Primary Endpoint
CLARITY-AD	Lecanemab	Placebo	898	897	18 months	CDR-SB
TRAILBLAZER-ALZ 2	Donanemab	Placebo	860	876	18 months	iADRS/CDR-SB
EMERGE	Aducanumab	Placebo	547	548	18 months	CDR-SB
ENGAGE	Aducanumab	Placebo	555	548	18 months	CDR-SB
GRADUATE I	Gantenerumab	Placebo	493	492	24 months	CDR-SB
GRADUATE II	Gantenerumab	Placebo	491	490	24 months	CDR-SB

GRADUATE consisted of two parallel phase III randomized trials (GRADUATE I and GRADUATE II), which were treated as independent randomized comparisons in the meta-analysis.

**Table 2:** Change in CDR-SB from baseline.

Trial	Mean Change (Active)	Mean Change (Placebo)	Mean Difference
CLARITY-AD	+1.21	+1.66	−0.45
TRAILBLAZER-ALZ 2	+1.72	+2.42	−0.70
EMERGE	+1.35	+1.74	−0.39
ENGAGE	+1.48	+1.62	−0.14
GRADUATE I	+2.34	+2.32	+0.02
GRADUATE II	+2.36	+2.33	+0.03

Legend. Change in Clinical Dementia Rating–Sum of Boxes (CDR-SB) from baseline in the included phase III randomized controlled trials. Positive values indicate worsening cognitive and functional impairment over the study period.

**Table 3:** Approximate amyloid plaque reduction reported in phase III anti-amyloid antibody trials.

Trial	Antibody	Approximate Amyloid Reduction
CLARITY-AD	Lecanemab	~60–70 centiloids
TRAILBLAZER-ALZ 2	Donanemab	~80–90 centiloids
EMERGE	Aducanumab	~50–60 centiloids
ENGAGE	Aducanumab	~40–50 centiloids
GRADUATE I/II	Gantenerumab	~20–30 centiloids

**Table 4:** Incidence of ARIA-E.

Trial	ARIA-E (%) Active	ARIA-E (%) Placebo	Risk Ratio
CLARITY-AD	12.6	1.7	7.4
TRAILBLAZER-ALZ 2	24.0	2.1	11.4
EMERGE	35.2	2.7	13.0
ENGAGE	34.0	3.1	11.0
GRADUATE I	28.8	1.9	15.1
GRADUATE II	27.5	2.0	13.7

Legend. Incidence of amyloid-related imaging abnormalities with edema or effusion (ARIA-E) in the included phase III randomized controlled trials. Percentages represent the proportion of participants experiencing ARIA-E in each treatment group during the randomized study phase.

## Data Availability

The original contributions presented in this study are included in the article/Supplementary Materials. Further inquiries can be directed to the corresponding author.

## Supplementary Materials

The following supporting information can be downloaded at: https://www.mdpi.com/article/10.3390/medsci14030337/s1, Table S1. Full-text studies excluded after eligibility assessment (*n* = 19) and reasons for exclusion according to PRISMA 2020 (Item 16b); File S2. PRISMA 2020 Checklist.

## References

[1] World Health Organization (2023). Dementia Fact Sheet.

[2] Hardy J., Selkoe D.J. (2002). The amyloid hypothesis of Alzheimer’s disease: Progress and problems on the road to therapeutics. Science.

[3] Van Dyck C.H., Swanson C.J., Aisen P., Bateman R.J., Chen C., Gee M., Kanekiyo M., Li D., Reyderman L., Cohen S. (2023). Lecanemab in early Alzheimer’s disease. N. Engl. J. Med..

[4] Sims J.R., Zimmer J.A., Evans C.D., Lu M., Ardayfio P., Sparks J., Wessels A.M., Shcherbinin S., Wang H., Monkul Nery E. (2023). Donanemab in early symptomatic Alzheimer disease. N. Engl. J. Med..

[5] Sevigny J., Chiao P., Bussière T., Weinreb P.H., Williams L., Maier M., Dunstan R., Salloway S., Chen T., Ling Y. (2016). The Antibody Aducanumab Reduces Aβ Plaques in Alzheimer’s Disease. Nature.

[6] Budd Haeberlein S., Aisen P.S., Barkhof F., Chalkias S., Chen T., Cohen S., Dent G., Hansson O., Harrison K., von Hehn C. (2022). Two randomized phase 3 studies of aducanumab in early Alzheimer’s disease. J. Prev. Alzheimer’s Dis..

[7] Avgerinos K.I., Ferrucci L., Kapogiannis D. (2021). Effects of Monoclonal Antibodies against Amyloid-β on Clinical and Biomarker Outcomes and Adverse Event Risks: A Systematic Review and Meta-Analysis of Phase III RCTs in Alzheimer’s Disease. Ageing Res. Rev..

[8] Sperling R.A., Jack C.R., Black S.E., Frosch M.P., Greenberg S.M., Hyman B.T., Scheltens P., Carrillo M.C., Thies W., Bednar M.M. (2011). Amyloid-related imaging abnormalities in amyloid-modifying therapeutic trials. Alzheimer’s Dement..

[9] Knopman D.S., Jones D.T., Greicius M.D. (2021). Failure to demonstrate efficacy of aducanumab: An analysis of the EMERGE and ENGAGE trials. Alzheimer’s Dement..

[10] Liu K.Y., Schneider L.S., Howard R. (2021). The need to show minimum clinically important differences in Alzheimer’s disease trials. Lancet Psychiatry.

[11] Canevelli M., Quarata F., Remiddi F., Sarti G., Grande G., Vanacore N., Bruno G. (2024). Anti-amyloid monoclonal antibodies for Alzheimer’s disease: A systematic review and meta-analysis. J. Alzheimer’s Dis..

[12] Page M.J., McKenzie J.E., Bossuyt P.M., Boutron I., Hoffmann T.C., Mulrow C.D., Shamseer L., Tetzlaff J.M., Akl E.A., Brennan S.E. (2021). The PRISMA 2020 Statement: An Updated Guideline for Reporting Systematic Reviews. BMJ.

[13] Higgins J.P.T., Thomas J., Chandler J., Cumpston M., Li T., Page M.J., Welch V.A. (2022). Cochrane Handbook for Systematic Reviews of Interventions.

[14] Jack C.R., Bennett D.A., Blennow K., Carrillo M.C., Dunn B., Haeberlein S.B., Holtzman D.M., Jagust W., Jessen F., Karlawish J. (2018). NIA-AA research framework: Toward a biological definition of Alzheimer’s disease. Alzheimer’s Dement..

[15] Albert M.S., DeKosky S.T., Dickson D., Dubois B., Feldman H.H., Fox N.C., Gamst A., Holtzman D.M., Jagust W.J., Petersen R.C. (2011). The diagnosis of mild cognitive impairment due to Alzheimer’s disease. Alzheimer’s Dement..

[16] Cummings J., Apostolova L., Rabinovici G.D., Atri A., Aisen P., Greenberg S., Hendrix S., Selkoe D., Weiner M., Petersen R.C. (2023). Lecanemab: Appropriate Use Recommendations. J. Prev. Alzheimer’s Dis..

[17] Khartabil N., Awaness A. (2025). Targeting Amyloid Pathology in Early Alzheimer’s: The Promise of Donanemab-Azbt. Pharmacy.

[18] Tolar M., Abushakra S., Hey J.A., Porsteinsson A., Sabbagh M. (2020). Aducanumab, Gantenerumab, BAN2401, and ALZ-801—The First Wave of Amyloid-Targeting Drugs for Alzheimer’s Disease with Potential for Near-Term Approval. Alzheimer’s Res. Ther..

[19] U.S. Food and Drug Administration (2020). Peripheral and Central Nervous System Drugs Advisory Committee Meeting: Aducanumab Briefing Document.

[20] European Medicines Agency (2023). Assessment Report: Lecanemab.

[21] Barcot O., Boric M., Poklepovic Pericic T., Cavar M., Dosenovic S., Vuka I., Puljak L. (2019). Risk of Bias Judgments for Random Sequence Generation in Cochrane Systematic Reviews Were Frequently Not in Line with Cochrane Handbook. BMC Med. Res. Methodol..

[22] Cumpston M.S., McKenzie J.E., Welch V.A., Brennan S.E. (2022). Strengthening systematic reviews in public health: Guidance in the Cochrane Handbook for Systematic Reviews of Interventions, 2nd edition. J. Public Health.

[23] Sterne J.A.C., Savović J., Page M.J., Elbers R.G., Blencowe N.S., Boutron I., Cates C.J., Cheng H.Y., Corbett M.S., Eldridge S.M. (2019). RoB 2: A revised tool for assessing risk of bias in randomised trials. BMJ.

[24] DerSimonian R., Laird N. (1986). Meta-analysis in clinical trials. Control. Clin. Trials.

[25] Higgins J.P.T., Thompson S.G., Deeks J.J., Altman D.G. (2003). Measuring inconsistency in meta-analyses. BMJ.

[26] Reiman E.M. (2016). Alzheimer’s Disease: Attack on Amyloid-β Protein. Nature.

[27] Honig L.S., Sabbagh M.N., van Dyck C.H., Sperling R.A., Hersch S., Matta A., Giorgi L., Gee M., Kanekiyo M., Li D. (2024). Updated safety results from phase 3 lecanemab study in early Alzheimer’s disease. Alzheimer’s Res. Ther..

[28] Zimmer J.A., Ardayfio P., Wang H., Khanna R., Evans C.D., Lu M., Sparks J., Andersen S., Lauzon S., Nery E.S.M. (2025). Amyloid-Related Imaging Abnormalities with Donanemab in Early Symptomatic Alzheimer Disease: Secondary Analysis of the TRAILBLAZER-ALZ and ALZ 2 Randomized Clinical Trials. JAMA Neurol..

[29] Mallinckrodt C., Tian Y., Aisen P.S., Barkhof F., Cohen S., Dent G., Hansson O., Harrison K., Iwatsubo T., Mummery C.J. (2023). Investigating Partially Discordant Results in Phase 3 Studies of Aducanumab. J. Prev. Alzheimer’s Dis..

[30] Bateman R.J., Smith J., Donohue M.C., Delmar P., Abbas R., Salloway S., Wojtowicz J., Blennow K., Bittner T., Black S.E. (2023). Two Phase 3 Trials of Gantenerumab in Early Alzheimer’s Disease. N. Engl. J. Med..

[31] Andrews J.S., Desai U., Kirson N.Y., Zichlin M.L., Ball D.E., Matthews B.R. (2019). Disease severity and minimal clinically important differences in clinical outcome assessments for Alzheimer’s disease trials. Alzheimer’s Dement..

[32] Vogel J.W., Young A.L., Oxtoby N.P., Smith R., Ossenkoppele R., Strandberg O.T., La Joie R., Aksman L.M., Grothe M.J., Iturria-Medina Y. (2021). Four Distinct Trajectories of Tau Deposition Identified in Alzheimer’s Disease. Nat. Med..

[33] Jack C.R., Andrews J.S., Beach T.G., Buracchio T., Dunn B., Graf A., Hansson O., Ho C., Jagust W., McDade E. (2024). Revised Criteria for Diagnosis and Staging of Alzheimer’s Disease: Alzheimer’s Association Workgroup. Alzheimer’s Dement..

[34] FDA (2021). FDA Grants Accelerated Approval for Alzheimer’s Disease Treatment.

