# Supplementary material for: Pharmacological and Clinical Heterogeneity of Anti-Amyloid Monoclonal Antibodies in Early Alzheimer’s Disease: A Systematic Review and Meta-Analysis of Randomized Trials
Source: Med Sci (Basel). 2026 Jun 23;14(3):337. doi: 10.3390/medsci14030337 (PMC13413489; doi:10.3390/medsci14030337)
Supplement: Supplementary file 1 [file medsci-14-00337-s001.zip › Supplementary File S2 ®C PRISMA 2020 Checklist.pdf]

PRISMA 2020 Checklist

Title:
Pharmacological and Clinical Heterogeneity of Anti-Amyloid Monoclonal Antibodies in Early Alzheimer's Disease: A Systematic Review and Meta-Analysis of Randomized Trials

TITLE

Item	Description	Location in Manuscript
1	Identify the report as a systematic review	Title

ABSTRACT

Item	Description	Location
2	Structured summary following PRISMA for Abstracts	Abstract section

INTRODUCTION

Item	Description	Location
3	Rationale for review	Introduction
4	Objectives	Study Rationale and Objectives

METHODS

Item	Description	Location
5	Eligibility criteria	Section 2.2
6	Information sources	Section 2.3
7	Search strategy	Section 2.3
8	Selection process	Section 2.4
9	Data collection process	Section 2.4
10a	Data items – outcomes	Section 2.2 and 2.4
10b	Other data items	Section 2.4
11	Risk of bias assessment	Section 2.5
12	Effect measures	Section 2.6
13a	Synthesis methods	Section 2.6
13b	Handling heterogeneity	Section 2.6
13c	Sensitivity analyses	Section 2.6
14	Reporting bias assessment	Section 2.6
15	Certainty assessment	Not performed (no GRADE assessment)
16	Registration and protocol	Section 2.1 (PROSPERO CRD420261323381)

RESULTS

Item	Description	Location
17	Study selection (numbers at each stage)	Section 3.1
18	Study characteristics	Section 3.2
19	Risk of bias results	Section 3.3

Item	Description	Location
20a	Results of individual studies	Tables 2 & 3
20b	Results of syntheses	Section 3.4 & 3.5
20c	Heterogeneity results	Section 3.4
21	Reporting bias	Section 2.6
22	Certainty of evidence	Not performed

DISCUSSION

Item	Description	Location
23a	Summary of evidence	Section 4
23b	Limitations of evidence	Section 4.5
23c	Limitations of review process	Section 4.5
23d	Implications	Section 4.4 & 4.6

OTHER INFORMATION

Item	Description	Location
24a	Registration	Section 2.1
24b	Protocol availability	PROSPERO
25	Funding	Funding section
26	Competing interests	Conflicts of Interest
27	Availability of data	Not applicable
