# Supplementary material for: Pharmacological and Clinical Heterogeneity of Anti-Amyloid Monoclonal Antibodies in Early Alzheimer’s Disease: A Systematic Review and Meta-Analysis of Randomized Trials
Source: Med Sci (Basel). 2026 Jun 23;14(3):337. doi: 10.3390/medsci14030337 (PMC13413489; doi:10.3390/medsci14030337)
Supplement: Supplementary file 1 [file medsci-14-00337-s001.zip › Supplementary_Table_S1_Excluded_Studies.pdf]

Supplementary Table S1

Full-text articles excluded after eligibility assessment (n = 19) with primary reason for exclusion, in accordance with PRISMA 2020 (Item 16b).

Supplementary Table S1. Full-text studies excluded after eligibility assessment (n = 19) and reasons for exclusion according to PRISMA 2020 (Item 16b)

Note: *The studies listed below are provided exclusively for PRISMA transparency as records excluded after full-text assessment. They are not considered references cited in the main manuscript and therefore are not included in the main reference list.*

No.	Excluded study / trial identifier	Therapeutic agent	Main reason for exclusion
**1**	Aducanumab Phase Ib (PRIME) trial	Aducanumab	Phase Ib study; not a randomized Phase III efficacy trial included in the quantitative synthesis
**2**	EMERGE Phase III trial	Aducanumab	Data already represented within pooled pivotal trial analyses; not included as an independent comparison according to eligibility criteria
**3**	ENGAGE Phase III trial	Aducanumab	Data already represented within pooled pivotal trial analyses; not included as an independent comparison according to eligibility criteria
**4**	Bapineuzumab Trial 302	Bapineuzumab	Population and outcome characteristics did not meet the predefined eligibility criteria
**5**	Solanezumab EXPEDITION 3 trial	Solanezumab	Did not fulfill the predefined criteria for inclusion in the comparative quantitative analysis
**6**	Crenezumab CREAD trial	Crenezumab	Trial design/population not compatible with the final eligibility criteria
**7**	Crenezumab CREAD 2 trial	Crenezumab	Trial design/population not compatible with the final eligibility

			criteria
**8**	BAN2401 Phase II trial	Lecanemab (BAN2401)	Phase II study; not included in the final randomized trial synthesis
**9**	CLARITY-AD Phase III trial	Lecanemab	Data not included as an independent study according to the predefined selection strategy
**10**	Donanemab Phase II (TRAILBLAZER-ALZ) trial	Donanemab	Phase II study; excluded from the final quantitative synthesis
**11**	TRAILBLAZER-ALZ 2 Phase III trial	Donanemab	Did not meet the predefined criteria for the final comparative dataset
**12**	Gantenerumab SCarlet RoAD trial	Gantenerumab	Early-phase study; not included in the final efficacy comparison
**13**	Gantenerumab Marguerite RoAD trial	Gantenerumab	Study population/design not compatible with predefined inclusion criteria
**14**	GRADUATE I trial	Gantenerumab	Data not included as an independent comparison according to the study selection strategy
**15**	GRADUATE II trial	Gantenerumab	Data not included as an independent comparison according to the study selection strategy
**16**	Solanezumab EXPEDITION trial program	Solanezumab	Earlier trial population not compatible with the final eligibility criteria
**17**	Solanezumab EXPEDITION 2 trial	Solanezumab	Did not meet the final selection criteria for quantitative synthesis
**18**	FDA regulatory briefing documents related to anti-amyloid antibodies	Multiple agents	Regulatory documents; not randomized clinical trials
**19**	EMA assessment reports related to anti-amyloid antibodies	Multiple agents	Regulatory assessment documents; not primary clinical trial reports
